# Greenland sharks keep their cool under fishing pressure

**DOI:** 10.1093/conphys/coag051

**Published:** 2026-07-25

**Authors:** Daniel M Ripley

**Affiliations:** Marine Biology Laboratory, Division of Science, New York University Abu Dhabi, Abu Dhabi, United Arab Emirates

Hidden under the Arctic ice lives the world’s oldest vertebrate, the Greenland shark (*Somniosus microcephalus*). Quietly patrolling beneath the waves, these “sleeper” sharks seldom come into contact with people—but what happens when they do? Fisheries in the Arctic target halibut but occasionally catch Greenland sharks instead. Owing to their exceptionally long lifespan, slow growth, and late sexual maturity, scientists believe that the Greenland shark is especially vulnerable to man-made stresses. As a primary bycatch species of commercial fisheries in the Arctic, this vulnerability is being tested. In the remote fjords of the Canadian Arctic, a team of researchers set out to test how stressful these accidental captures can be, finding that when handled correctly, these ancient sharks remain remarkably cool under pressure (Watanabe *et al.*, 2026).

**Figure 1:**
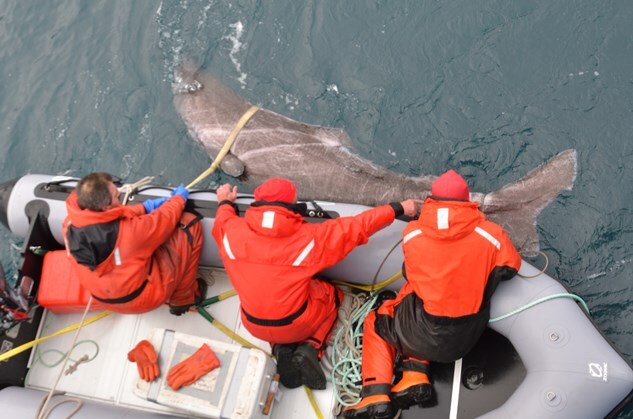
Capture and sampling of a Greenland shark. Image credit: Yuuki Watanabe

To determine how stressful being caught and released is, Yuuki Watanabe and colleagues caught 12 Greenland sharks (Fig. 1), took small blood samples, and equipped them with Global Positioning System trackers to monitor their behaviour and survival before releasing them back into the wild. By doing so, the scientists were able to estimate stress levels in the sharks, as well as track their location, speed, and survival after being released.

During capture, Greenland sharks had lower levels of lactate and glucose in their blood—molecules associated with exhaustive exercise and physiological stress—than many other species of shark. Once released, the sharks swam hard and fast (for a Greenland shark), with their tail-beat frequency and swimming speed being higher than baseline levels. However, their behaviour returned to normal within ~10 hours, a timeframe similar to that of other shark species. In the 30 days following release, when most capture-related mortality is observed, survival was high (≥90%), further demonstrating their resilience.

But what helps this ocean elder remain cool under pressure? The researchers believe that the shark’s calm demeanour, characterised by its minimal fight response and slow pace of life, may be the answer. Many species fight for hours when hooked, exhausting themselves and increasing their physiological stress levels. However, these “sleeper” sharks may adopt a more relaxed approach, with a laidback lifestyle and subdued fight response. This theory was supported by the researchers observing no correlation between hooking duration and lactate concentrations in the shark’s blood. Additionally, the Arctic environment may help. Warm surface waters, and large temperature differences between the surface and deeper habitat, are known to increase stress during capture. In the Arctic, surface waters are cool (~3°C) and only slightly warmer than the shark’s typical habitat, so this small temperature change may further minimise stress. These results highlight that, when handled appropriately, Greenland sharks remain calm under pressure, quickly returning to normal behaviour and showing high survival rates after catch-and-release.
